# Functional roles of the microbiota-gut-brain axis in Alzheimer’s disease: Implications of gut microbiota-targeted therapy

**DOI:** 10.1515/tnsci-2020-0206

**Published:** 2021-12-28

**Authors:** Si-Ran Zhong, Qi Kuang, Fan Zhang, Ben Chen, Zhen-Guo Zhong

**Affiliations:** School of Health Medicine, Guangzhou Huashang College, Guangzhou, 511300, People’s Republic of China; International Institute for Translational Chinese Medicine, Guangzhou University of Chinese Medicine, Guangzhou, 510006, People’s Republic of China; Scientific Research Center of Traditional Chinese Medicine, Guangxi University of Chinese Medicine, Nanning City, 530200, Guangxi Zhuang Autonomous Region, People’s Republic of China

**Keywords:** Alzheimer’s disease, microbiota-gut-brain axis, gut microbiota, blood–brain barrier, lipopolysaccharides, short-chain fatty acids, oligosaccharides, microbial amyloid, enteric nervous system, *Bacteroides*

## Abstract

Increasing scientific evidence demonstrates that the gut microbiota influences normal physiological homeostasis and contributes to pathogenesis, ranging from obesity to neurodegenerative diseases, such as Alzheimer’s disease (AD). Gut microbiota can interact with the central nervous system (CNS) through the microbiota-gut-brain axis. The interaction is mediated by microbial secretions, metabolic interventions, and neural stimulation. Here, we review and summarize the regulatory pathways (immune, neural, neuroendocrine, or metabolic systems) in the microbiota-gut-brain axis in AD pathogenesis. Besides, we highlight the significant roles of the intestinal epithelial barrier and blood–brain barrier (BBB) in the microbiota-gut-brain axis. During the progression of AD, there is a gradual shift in the gut microbiota and host co-metabolic relationship, leading to gut dysbiosis, and the imbalance of microbial secretions and metabolites, such as lipopolysaccharides (LPS) and short-chain fatty acids (SCFAs). These products may affect the CNS metabolic state and immune balance through the microbiota-gut-brain axis. Further, we summarize the potential microbiota-gut-brain axis-targeted therapy including carbohydrates, probiotics, dietary measures, and propose new strategies toward the development of anti-AD drugs. Taken together, the data in this review suggest that remodeling the gut microbiota may present a tractable strategy in the management and development of new therapeutics against AD and other neurodegenerative diseases.

## 1 Introduction

Alzheimer’s disease (AD) is the most common form of dementia, affecting 50 million people across the world. Between 2000 and 2013, deaths as a result of cerebrovascular disease decreased by 20%, whereas AD mortality increased by 71% [1].

The most prevalent clinical manifestation of AD is progressive memory deficit with multiple cognitive impairments. Based on the “amyloid cascade” hypothesis, AD results from amyloid-β (Aβ) deposition and neurofibrillary tangles. They are characterized by hyperphosphorylated tau protein and neuronal loss in multiple brain sections including the hippocampus, neocortex, amygdala, and the nucleus basalis of Meynert [2,3]. However, the exact cause of AD remains elusive. Despite the fact that many patients with mild cognitive impairment show no Aβ deposits in the brain, some nondemented elderly people have Aβ deposits or senile plaques [4]. In recent years, more than 20 anti-AD compounds that target Aβ or tau aggregation have shown no efficacy in slowing cognitive deficit [5], raising a great challenge to the “amyloid cascade” hypothesis.

On the other hand, neuroinflammation theory suggests that chronic inflammation characterized by microglia activation and innate immune response both in the brain and in the periphery mediates AD progression [6].

Microglia is the key player in this process. It is involved in AD pathogenesis by releasing inflammatory mediators, manifested as a major regulator of inflammation, NF-κB dysregulation, resulting in Aβ accumulation and oxidative stress.

More importantly, Aβ itself is a microglia activator. This cycle has been considered as an underlying factor in AD pathogenesis [7,8]. Persistent neuroinflammatory and astrocytes activation contributes to the blood–brain barrier (BBB) dysfunction, and thus, to neuronal death and cognitive impairment. Whereas huge efforts have been made towards the development of new drugs targeting inflammation and immune regulation [9,10,11], the options for clinical treatment remain limited [12]. Nevertheless, this reasoning led to the development of the amyloid hypothesis and subsequent pharmacology development, which also faced tremendous challenges. Therefore, the need for the development of new therapeutics against AD remains alive.

In the last decade, the human microbiome project has expanded our understanding of the human microbiome and disease development [13,14,15,16,17]. The intestinal microorganisms encode 4 × 10^6^ genes, which is about 150× higher than humans [13,15], which ultimately contributes to the sophisticated microbial diversity. A plethora of evidence has suggested that dysbiosis of gut microbiota in human beings is closely associated with many illnesses, especially metabolic-related diseases [18,19,20] (insulin resistance Type 2 diabetes, obesity, and liver diseases), cardiovascular diseases [21], or malnutrition [22].

A growing number of studies have demonstrated variations in the composition of gut microbiota, which contribute to the alteration in brain function and behavior, a phenomenon referred to as the microbiota-gut-brain axis [23,24,25].

Studies based on germ-free (GF) animals [26], antibiotics [27], probiotics [28], or fecal microbiota transplantation [29] support the pivotal role of gut microbiota in modulating cognition, behavior, and central nervous system (CNS) physiology. Whereas there is evidence that gut microbiota affects brain physiology and Aβ accumulation and even AD, its regulatory role in AD still remains elusive. A newly developed drug, GV-971, has been shown to work by regulating gut microbiota dysbiosis, which elucidated a novel way in the development of new anti-AD drugs [30].

In this review, we summarize the gut microbiota alteration in crosstalk between the gut and the brain, mediated by the microbiota-gut-brain axis in AD pathogenesis. We highlight the significance of gut microbiota in maintaining host metabolic and immune homeostasis, as well as a potential therapeutic target for neurodegenerative diseases and AD. Furthermore, we discuss recent research on AD therapy advances, especially in remodeling the gut microbiota. We highlight the potential drug candidate targeting the microbiota-gut-brain axis that may define the discovery of novel therapeutic agents.

## 2 Gut ecosystem and dysbiosis in AD

Gut microbiota is a symbiotic system having bacteria, viruses, and fungi in the human gut, which affect human digestion, intestinal biosynthesis, metabolism, and inflammation [31,32]. There are up to 1,000 microbial species that are composed of 90% *Firmicutes* and *Bacteroidetes*, and 10% *Actinobacteria*, *Proteobacteria*, *Fusobacteria*, and *Verrucomicrobia*. Like in the legume-rhizobia symbiotic relationship, gut microbiota and the human body is unique ecosystem and is an outcome of biological evolution. Recently, a cohort study of around 2,100 gut microbiota metagenomes uncovered over 22.3 million nonredundant prokaryotic genes, with half of the genes being unique in a single person [33]. Collectively, the gut microbiota is a highly active, extremely dynamic, and vast heterogenetic ecosystem, which is defined by the host’s age, disease status, diet, environment, and ethnicity [34].

The gut microbiota ecosystem is beneficial to human health in several ways:

(1) Biosynthesis of metabolic co-factors, polysaccharides, and fatty acids to supplement host nutrition [35,36];
(2) Metabolism of products such as cleavage of dietary fiber into short-chain fatty acids (SCFAs), which can modulate intestinal epithelial barrier (IEB) permeability, maintain homeostasis of the host immune system and glucose homeostasis [37,38]; On the other hand, microbiota and its metabolism can drive immune functions, which is characterized by cytokine signal release, and consequently, alter the activation status and physiology of neurons and glial cells [39].
(3) Provision of protection against pathogens, as well as maintenance of the homeostasis of the microbiota composition [40,41]. On the contrary, when the IEB is dysfunctional, the secretory neurotoxins or metabolites of enterotoxigenic strains can leak through the barrier causing substantial inflammatory pathology or metabolic diseases.
(4) The neuroendocrine system regulated by microbiota can modulate neurotransmitter production, such as 5-hydroxytryptamine and tyramine. The neurotransmitter production in the intestine and delivery to the brain may play a central role in brain neurodevelopment [42]. Meanwhile, the hypothalamic–pituitary–adrenal (HPA) axis form the bidirectional communication between the gut microbiota and the brain [43]. Together, the neuroendocrine system can maintain the integrity of the immune system, IEB, and BBB.

More importantly, through these ways, gut microbiota can regulate neurological outcomes and maintain the behavior and development of neural and immune systems [44,45].

GF animals are microbial-deficient animals, in a sterile environment. These animals offer unique opportunities to explore how the gut microbiota participate in AD pathogenesis. The GF mice showed deficits in the nonspatial task (object recognition test) and reduced brain-derived neurotrophic factor (BDNF) in the hippocampus [46]. On the other hand, the GF mice exhibited innate immune response impairment, manifested as microglial immaturity. Whereas the GF amyloid precursor protein (APP)/PS1 mice showed a reduction in Aβ pathology, the cerebral Aβ were rescued by fecal microbiota transplantation from conventionally raised APP/PS1 control mice [26]. Fujii et al. demonstrated that mice transplanted with gut microbiota from a patient with AD exhibited behavior and cognition dysfunction [47].

To identify specific microbiota shifts, several studies have performed 16S rRNA sequencing in APP/PS1 transgenic mice and AD patients (Table 1). Those results highlight the significant effect of *Bacteroides fragilis* in cognitive impairment and brain amyloidosis [48,49,50]. From the data, we inferred that gut microbiota may participate in AD pathogenesis.

**Table 1:** Summary of intestinal microbiota change in AD

Fecal sample source	Altered gut microbiota (AD versus control)		References
	Upregulated	Downregulated	
AD patients	*Bacteroidetes*, *Escherichia/Shigella*, *Ruminococcus*	*Firmicutes, Bifidobacterium, Eubacterium rectale, Lachnospiraceae, Selenomonadales*	[48,49,50]
APP/PS1 transgenic mice	*Bacteroidetes*, *Tenericutes*	*Firmicutes, Verrucomicrobia, Proteobacteria, Actinobacteria, Allobaculum, Akkermansia*	[26]

## 3 The function of intestinal–blood barrier and brain–blood barrier in the AD microbiota-gut-brain axis

The single-layer epithelial cells make up the mucosal interface, which separates the host and microorganisms, allowing selective small compounds to permeate, thus limiting the access of pathogens and metabolic antigens, forming the IEB. The IEB contains a plethora of immune effector cells, which provide physiological and defense support to the host. The permeability and functions of the IEB are mainly modulated by substances in the tract, such as microbiota, immune cells, inflammatory substances, or metabolites. More recently, the concept gut-vascular barrier (GVB) was proposed [51], which is a cellular barrier below the epithelium. GVB is the second layer of defence, which controls the antigens translocation and prohibits the entry of the gut microbiota.

The BBB is composed of brain endothelial cells and pericytes, compartmentalized peripheral circulation, and CNS. Intestinal microbiota dysbiosis resulting in the alteration of the IEB permeability, referred to as the leaky gut, leads to the leakage of microbial metabolites or secretion (endotoxins like lipopolysaccharide) and peripheral circulation inflammation. The inflammation triggers activation of peripheral monocytes that cross the BBB leading to the production of inflammatory cytokines and microglia activation, which ultimately results in neuroinflammation in AD. Moreover, peripheral immune cell infiltration, such as 17 T-helper cells, plays an essential role in neuroinflammation [25].

Both the IEB and BBB play an essential role in the intestinal tract, peripheral circulation, and CNS. They limit the infiltration of harmful substances and have a significant effect on immune recognition and maintenance of homeostasis. In addition, the IEB and BBB are vital pathway regulators in the microbiota-gut-brain axis.

## 4 Regulation pathway in the AD microbiota-gut-brain axis

**Figure 1:**
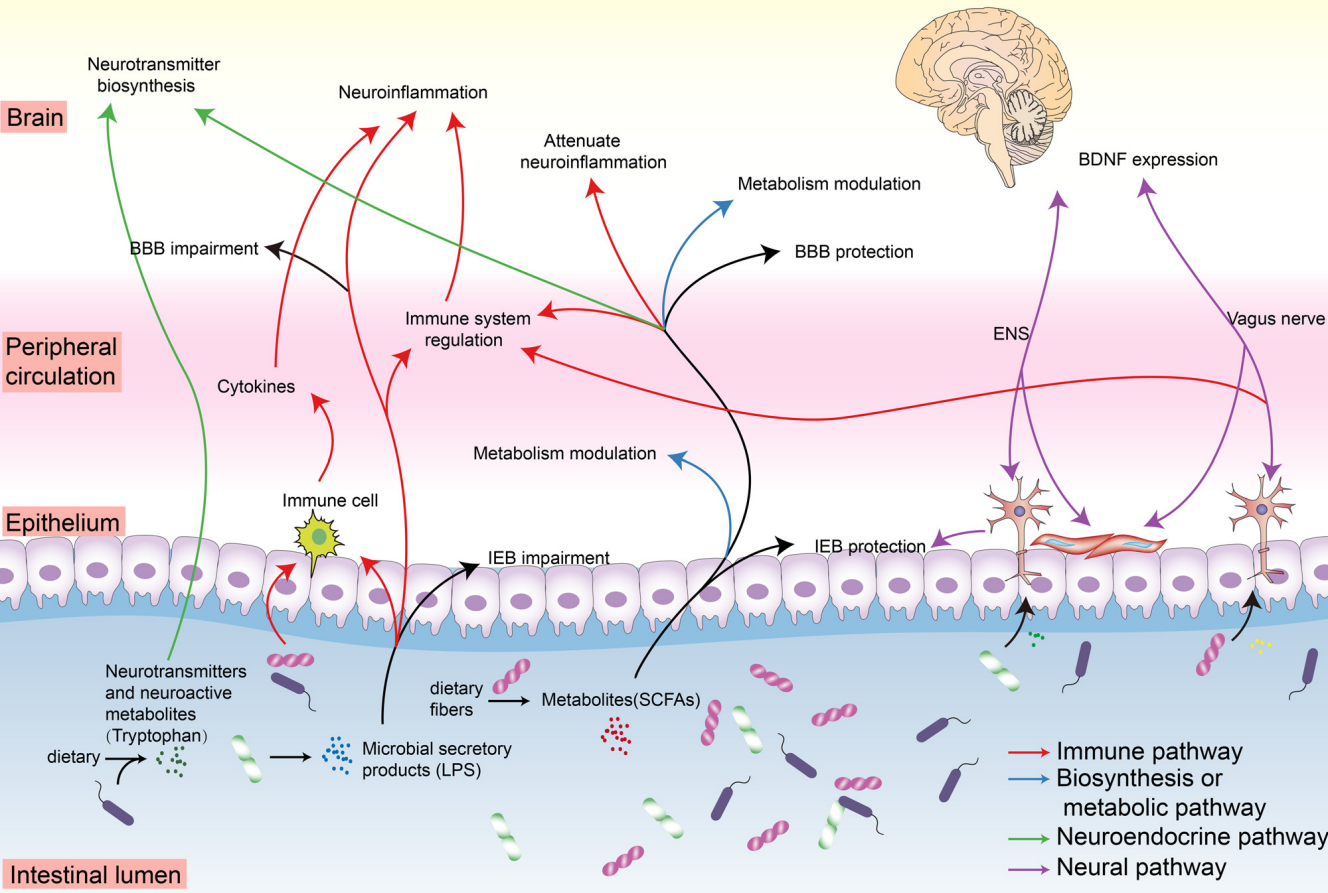
The microbiota-gut-brain axis regulatory pathways involved in AD pathology. An outline illustrating several important regulatory pathways between gut microbiota and the brain, including immune pathway, neural pathway, neuroendocrine pathway, biosynthesis or metabolism pathway, and their interaction relationship (Table 2). Microbial secretory products, such as LPS, play a key role in immune activation in the gut, periphery, and brain, which contribute to neuroinflammation. Neurotransmitters and neuroactive metabolites secreted by microbiota can interact with host neurotransmitter signaling and synthesis. Neural pathways including ENS and vagus nerve play a vital role in regulating gut physiologic and brain cognition behavior. Some microbial metabolites, such as SCFAs, have an important neuroprotective effect against neuroinflammation in the brain, and regulatory effects in modulating host metabolism and immunity. IEB and BBB functionality also play a significant role in host homeostasis. BDNF, brain-derived neurotrophic factor; ENS, enteric nervous system; LPS, lipopolysaccharides; IEB, intestinal epithelial barrier; BBB, blood–brain barrier; SCFAs, short-chain fatty acids.

Potential pathways of gut microbiota affect CNS through the microbiota-gut-brain axis including the following aspects (Figure 1):

(1) The gut microbiota composition can be influenced by daily diet preference, exogenously administered probiotics [52], potential bacteria, and medication. For example, the microbiota can compete for nutrients as growth substrates, produce fermentation products, compete for binding sites on the enteric wall, and reduce inflammation [24,52], which is the basis for the microbiota-gut-brain axis signaling.
(2) Metabolites or secretory products, such as short-chain fatty acids and lipopolysaccharides, produced by gut microbial fermentation of fibers can interact with epithelial cells and immune cells on the mucosal interface. After that, it can be transported or diffused into host cells and peripheral circulation, and mediate cytokine secretions, innate immune or adaptive immune response, and peripheral inflammation [53]. The metabolites can further reach the brain and participate in neuroinflammation. These microbiota-gut-brain pathways participated in the gut-periphery-brain immune cascade reaction and are referred to as the immune pathway.
(3) Apart from that, some metabolites can participate in enzyme biosynthesis, host metabolism, and bioactive signaling, resulting in the modification of energy supplement metabolism or neuroepigenetic modulation [54].
(4) Gut microbiota can directly produce a vast array of neuroactive compounds that participate in neurotransmitter signaling [55]. For instance, *Lactobacillus* and *Bifidobacterium* can directly produce γ-aminobutyric acid (GABA) [56], while *Escherichia* and *Serratia* are associated with dopamine production. Besides, the gut microbiota is also able to indirectly take control of the neurotransmitter biosynthesis by modulating neurotransmitter precursors of neuroactive compounds [57]. Those directives and indirective ways for host and microbiota communication in the microbiota-gut-brain axis are called the neuroendocrine pathway.
(5) Gut microbiota and its metabolites can affect brain cognition and behavior by vagus nerve or enteric nervous system (ENS) stimulation [58,59]. Meanwhile, activation of the vagus nerve has shown anti-inflammatory ability against microbial-induced sepsis through the nicotinic acetylcholine receptor, which highlights the role of the neural immunomodulation system [60].

These regulatory pathways play their role not only independently, but also collaboratively or interactively in the microbiota-gut-brain axis. For example, microbiota-neuroendocrine immunomodulation and neural immunomodulation effect in the microbiota-gut-brain axis were elucidated in several studies [61,62].

In the following section, we will discuss in detail how the gut ecosystem alteration contributes to AD pathogenesis through the microbiota-gut-brain axis regulatory pathway.

### 4.1 Microbiota metabolism modulation and its neuroendocrine role in the AD microbiota-gut-brain axis

Gut metabolites are modified by the host and the microbiota. This co-metabolic mode is an essential part of human metabolic and immune homeostasis. Microbiota metabolism can also directly regulate the neurotransmitter biosynthesis through the neuroendocrine pathway directly. Meanwhile, the generated metabolites in the intestinal tract are diffused into the host body, thus modulating the BBB and affecting the innate immune activation, which indirectly affects the microbiota-gut-brain axis. Here, we focus on specific co-metabolism alteration and the neuroendocrine role, which contribute to the AD pathogenesis in the microbiota-gut-brain axis.

The microbiota metabolism can modulate the neurotransmitters or their precursor biosynthesis and consequently affect the microbiota-gut-brain axis through the neuroendocrine pathway. Tryptophan is a central amino acid precursor to 5-HT synthesis and other aminergic neurotransmitters serotonin. Meanwhile, it cannot be synthesized by the human body, but the gut bacteria are able to produce tryptophan through the shikimate pathway. The metabolism and catabolism of tryptophan affect several neuroactive compounds. More importantly, most bacterial strains harbor the tryptophanase enzyme, an enzyme that metabolizes tryptophan. Tryptophanase from *Escherichia coli* generates indole by tryptophan catabolism [63]. Hence, the metabolism of tryptophan modulates the brain neurotransmitter signaling through the microbiota-gut-brain axis. The kynurenine pathway (KP), on the other hand, is a major platform for tryptophan catabolism in the peripheral and CNS. The altered KP in the peripheral system was observed in AD patients [64]. The tryptophan catabolism enzymes such as hepatic tryptophan 2,3-dioxygenase and indoleamine 2,3-dioxygenase (IDO) are key modulators in the neuroendocrine pathway. The KP can be upregulated by the cytokine-mediated innate immune system, in which interferon-γ activates the IDO, an enzyme for kynurenines catalysis. Kynurenine/tryptophan ratio (Kyn/Trp) is an index for tryptophan breakdown or KP activity. It is associated with not only immune activation but also cognitive performance in AD [65,66]. A larger community-based cohort study also revealed that, unlike tryptophan, the kynurenine levels increase with age [67]. Collectively, tryptophan and KP may contribute to AD pathogenesis mediated by the neuroendocrine pathway (Table 2).

**Table 2:** Summary of regulatory factors in the microbiota-gut-brain axis involved in AD pathology

Category	Regulator in the microbiota-gut-brain axis	Associated microbes	Associated regulatory pathway	Effect on barrier	Regulatory effect	References
Microbial secretory product	LPS	*Bacteroides fragilis*	Immune pathway;	IEB and BBB impairment	NF-κB (p50/p65) activation; stimulation of the innate immune system	[86,90,93]
	Microbial amyloid	*Escherichia coli*; *Salmonella enterica*	Immune pathway; neural pathway	/	NF-κB (p50/p65) activation; amyloid proteins cross-seeding; stimulation of the innate immune system; Vagus nerve	[92,102,105]
	PSA	*Bacteroides fragilis*	Immune pathway; neural pathway	/	T regulatory cells migration to CNS	[112,113]
Microbial metabolism related produce	SCFAs	*Ruminococcus bromii* (butyrate); *Faecalibacterium prausnitzii*, *Eubacterium rectale*, *Eubacterium hallii* and *R. bromii* (major fraction of butyrate production); *Akkermansia municiphilla* (propionate)	Neuroepigenetic modulator and signaling molecule; biosynthesis or metabolic pathway; immune pathway; neuroendocrine pathway	IEB and BBB protection	Intertion of neurotransmitter biosynthesis; HDACs inhibitors; GPCR activator; NF-κB inactivation; downregulation of pro-inflammatory cytokine TNF	[66,67,68,69,70]
	Tryptophan and KP	/	Neuroendocrine pathway; neuroendocrine-immune pathway	/	KP; aminergic neurotransmitter serotonin; inductor of innate immune system activation and infammation	[64,65]
Microbiota related nerve	ENS	/	Neural pathway	IEB protection	Regulator of IEB permeability and function	[117,120]
	vagus nerve	/	Neural pathway; Vagal immunomodulation pathway	/	Modulation of anxiety-like behavior; increased BDNF expression	[58,110]

GPCR, G protein-coupled receptors; HDACs, histone deacetylases; TNF, tumor necrosis factor; IEB, intestinal epithelial barrier; BBB, blood–brain barrier; BDNF, brain-derived neurotrophic factor.

SCFAs, including propionic acid, acetic acid, and butyric acid, are produced from undigested carbohydrates or dietary fiber, or complex polysaccharides by gut microbiota. The concentrations of SCFAs are affected by the microbiota composition and abundance, dietary fiber content, and metabolic flux of SCFAs. A comprehensive SCFA study revealed that the contents of propionic acid, isobutyric acid, and 3-hydroxybutyric acid (butyric acid) significantly decreased in AD transgenic mice fecal samples compared to the wild type [68]. Meanwhile, a recent study found dysregulation of gut microbiota correlated with the change in the SCFA level [69], and SCFAs could inhibit Aβ aggregation *in vitro* [70]. Those results support that SCFAs may play a vital role in AD pathogenesis. But how do SCFAs participate in AD pathogenesis? It is well established that SCFAs play a vital role in maintaining the IEB and BBB structural integrity. The IEB protective effect of SCFAs is mainly through induction of immune tolerance, activation of inflammasomes, and interleukin-18 production. Recent studies showed that SCFAs have a protective effect on IEB against a fructose diet-induced hippocampal neuroinflammation and neuronal impairment [71], and propionate can protect the BBB from oxidative stress [72].

Furthermore, studies have demonstrated that sodium butyrate not only exerts a neuroprotective effect via restoring the BBB [73] and IEB functionality [71] but also improved memory function in the AD model mice via the inhibition activity of histone deacetylases (HDACs) [74,75]. SCFAs demonstrate as HDAC inhibitors and G protein-coupled receptor (GPCR) activator, involved in NF-κB inactivation [54] and downregulation of the pro-inflammatory cytokine tumor necrosis factor (TNF) [75,76]. By inhibiting HDACs, it was revealed that butyrate and propionate can suppress adaptive immune activity by suppressing the expression of T cell-activating proteins on antigen-presenting cells (APCs) [77]. The immune modulation effect of SCFAs on APCs also plays an important role in neuroinflammation. Additionally, HDAC2 has been demonstrated as an epigenetic regulator involved in synaptic plasticity deficit and cognitive impairment attributed to AD pathogenesis [78]. Frost et al. revealed that acetate can regulate central hypothalamic metabolism and transcellular neurotransmitter biosynthesis cycles [79]. It participates in the tricarboxylic acid cycle in astrocyte and GABA neuroglial biosynthesis in neurons, resulting in reduced appetite. In their study, they highlighted that SCFAs interact with brain metabolism and neurotransmitter biosynthesis (Table 2).

By fecal microbiota transplantation in APP/PS1 transgenic AD model mice, researchers demonstrated that the change in SCFAs is restored along with cognitive deficit and synaptic plasticity [80]. Meanwhile, Wenzel et al. demonstrated that formate could reduce phagocytic activity, inhibit respiratory burst, and reduce the reactive oxygen species (ROS) production *in vitro* [81]. This study proposed that SCFAs have a modulation effect on disrupted microglial that participate in AD neuroinflammation. On the other hand, gut microbiota or microbial metabolites including SCFAs have shown a detrimental role in another neurodegenerative disease Parkinson’s disease (PD), while antibiotic treatment ameliorates pathophysiology and neuroinflammation in PD animals [82]. This controversial result prompts us to rethink the role of microbiota in neurodegenerative diseases. Collectively, microbiota metabolites including SCFAs, generated and modified by gut microbiota, play a vital role in IEB and BBB modulation and affect the AD microbiota-gut-brain axis, but a controversial role in PD [83]. Hence, through modulating the microbiota composition and metabolites, the IEB and BBB may be viable targets in the microbiota-gut-brain axis. More importantly, by acting as a neuroepigenetic modulator and signaling molecule, SCFAs can regulate immunity and metabolism in the gut or in the host body. This provides us with an intervention approach for reshaping the gut microbiota through dietary ways, which will be discussed in the following part.

The HPA axis is another major regulatory process in the neuroendocrine pathway that closely interacts with the gut microbiota and the brain. Corticotropin-releasing factor, adrenocorticotropic hormone, and glucocorticoids are three principal regulators of the HPA signaling axis [43]. The HPA axis is closely associated with brain function and anxiety or depression-related behaviors by interacting with the immune system, IEB, and BBB [84]. However, a few studies reported the exact mechanism underling the HPA axis in the AD microbiota-gut-brain axis.

### 4.2 Microbial secretory products in the AD microbiota-gut-brain axis

Gut microbiota plays a vital role in regulating the innate immune system due to the abundance of foreign antigens [85]. Germline-encoded pattern recognition receptors (PRRs) is a vital compartment in the innate immune system as it helps the host to distinguish self and nonself antigens, and respond to changes in the microbial landscape. PRRs can recognize bacterial, fungal, and its associated secretory products such as lipopolysaccharides (LPS), polysaccharide A (PSA), and unique nucleic acid structures by monitoring microorganism-associated molecular patterns (MAMPs) or pathogen-associated molecular patterns. Here, we focus on how gut microbiota and its secretory products interact with the innate immune system. This interaction results in the production of AMPs, cytokines, or chemokines, as well as systematic circulation inflammation and barrier impairment, which ultimately lead to neuroinflammation and thus AD pathogenesis.

*Bacteroides* is the largest phylum of Gram-negative bacteria in the intestinal tract microbiome, which secretes a complex array of pro-inflammatory neurotoxins, and its content correlates with inflammatory signaling and permeability of the BBB. *Bacteroides fragilis (B. fragilis)* is a nonspore forming, nonmotile, commensal, and obligatory anaerobic bacillus in the human intestines with multiple capabilities for human health maintenance. The secretory products of *B. fragilis* are highly related to the immune pathways in the microbiota-gut-brain axis.

LPS are a major component of the Gram-negative bacteria cell wall. It is composed of a highly immunogenic class of amphipathic surface glycolipids. LPS can interact with Toll-like receptors (TLRs) by its MAMPs, resulting in innate immune activation and so is often used for inflammation inductor in experimental animal models. LPS can be excreted by Gram-negative bacteria in the human gut and may contribute to the pathogenesis of AD during aging. It has been reported that chronic infusion of LPS into the rat brains successfully reproduced inflammatory and pathological changes similar to AD patients [86], and the Aβ1-42 levels significantly increased in the hippocampal tissue [87]. How does the LPS in the intestine tract influence CNS inflammation? LPS is also transported from the intestine tract to CNS in a gut-periphery-brain manner, resulting in neuroinflammation, which refers to this process as the immune pathway. In the AD pathological condition, the amyloid-beta 42 (Aβ42) peptide can facilitate the entry of LPS into neurons and nuclei [88]. Those studies suggest that LPS may induce neuroinflammation and further result in Aβ accumulation that contributes to AD pathology.

In CNS, LPS can downregulate pre- and post-synaptic proteins and impair the efficient readout of neuronal genetic information in primary human neuronal-glial cell co-culture experiments, which reveals that the CNS synaptic structure and neurotransmission is compromised in LPS-induced neuroinflammation [89,90]. LPS could be recognized by TLR 4 on the microglial depending on the cluster of differentiation 14 (CD14) expression, further resulting in microglial activation and immune cell infiltration [91]. Also, CD14 expression plays a vital role in Aβ clearance [91].

On the other hand, LPS secreted by the *B. fragilis*, called BF-LPS, can impair biophysiological barriers via cleavage of intercellular proteins, inducing leaky gut, causing LPS. A recent study reported that microbiome-derived BF-LPS levels from the hippocampus and superior temporal lobe neocortex of AD were significantly increased [92,93]. BF-LPS is demonstrated as an exceptionally potent inducer of NF-κB (p50/p65) [94], which is a pro-inflammatory transcription factor, and subsequently, induces pro-inflammatory microRNAs release, such as miRNA-9, miRNA-125b, and miRNA-155 [95,96,97]. Ultimately, the microRNAs can further downregulate the SH3-proline-rich multidomain-scaffolding protein (SHANK3), the triggering receptor in microglial cells (TREM2), and complement factor H [95,97]. This BF-LPS induced NF-κB-mediated pro-inflammation-miRNA signaling may be a potential contributor to AD neuroinflammation. More recently, Yang et al. demonstrated that probiotics could decrease the LPS concentration and the corresponding NF-κB signaling pathway in the AD model mice [98]. The oral treatment with probiotics *Bifidobacterium longum* (*B. longum*) also can suppress gut dysbiosis NF-κB activation induced by LPS [99].

These data suggest that LPS may induce neuroinflammation by microglia activation and highlight the potential role of BF-LPS induced NF-κB-mediated inflammation pathway in the microbiota-gut-brain axis. On the other hand, probiotics exhibit a potent protective effect against microbe secreted neurotoxins. This leads to opportunities for probiotic intervention, in which we could rebalance the phylum or microbiota composition in the gut to achieve a balance immune crosstalk between brain and gut.

Alpha synuclein (α-syn), amyloid β (Aβ), tau, and TAR DNA-binding protein 43 are amyloid proteins with prion-like features, and their deposition in the CNS may trigger neurodegenerative disorders [100]. The classical “amyloid cascade” hypothesis in AD suggests that the aggregation of Aβ is a characteristic pathological feature for AD progression. The expression of APP, a major factor in the Aβ biosynthesis, was found to modulate intestine immune phenotype and immune-related disorders. Intriguingly, the gut microbiota is a major source of secretory amyloids. In contrast to amyloid proteins produced by mammals, curli proteins are functional amyloid fibers produced by *Escherichia coli* (*E. coli*) during biofilm formation and colonization [101].

Moreover, abundant evidence has demonstrated that microbial amyloids are capable of eliciting cross-seeding or transiently interacting with host amyloidogenic proteins (tau, Aβ, α-syn, and prion) and affect aggregation [102,103,104]. Different forms of amyloid proteins can accelerate the amyloidogenesis of heterologous amyloid proteins [105,106]. Chen et al. demonstrated that oral administration of curli can accelerate α-syn aggregation and increase its deposition in the gut and the brain, priming the innate immune system and neuroinflammation in the CNS [107]. Similarly, Sampson revealed that microbial amyloid subunit of curli (CsgA) can exacerbate aSyn pathology in the gut and the brain [108]. In addition, curli cross-seeding with Aβ was also observed, indicating that amyloid cross-seeding may be mediating AD pathology [109].

On the other hand, microbial amyloids can be recognized by TLRs 2 as a pathogen due to their structural similarity with Aβ, a phenomenon referred to as molecular mimicry. This results in the priming of the innate immune system, manifested as interaction with CD14, activation of NF-κB, thus leading to neuroinflammation and Aβ aggregation [109]. It has been shown that TLR2, Il-6, and TNF are upregulated in the brain of the animals exposed to curli [107]. By tracking the spread of the pathologic amyloid across different brain sections, Kim et al. demonstrated the capability of microbial amyloid to spread from the gut to the brain via the vagus nerve [110]. Moreover, another study revealed that amyloid proteins in the CNS can transfer to distant organs, such as the stomach by motor vagal projection [111]. The two studies revealed the amyloid proteins’ metastatic ability and showed that the microbial amyloids or microbial secretory products could act as bidirectional communication massagers, thus highlighting the potential of the microbiota-gut-brain axis. However, these studies mainly focused on the role of α-syn in PD. The data on the effect of Aβ on the gut microbial and host amyloidogenesis process remain scant. Collectively, microbial amyloid is able to bidirectionally communicate to the brain via the amyloid proteins cross-seeding, stimulation of the innate immune system, and bidirectional communication via the autonomic or vagus nerve.

PSA is a capsular polysaccharide produced by *B. fragilis*. PSA is vital for the growth and colonization of *B. fragilis*. PSA has modulatory effects on the innate and adaptive immune system through the TLR-2, which affects AD neuroinflammation [112,113]. A study showed that oral administration of PSA can promote the expression of immune-regulatory CD4^+^ T cells CD39, enhancing the migration of T regulatory cells (Treg) to the CNS. This cascade of events reveals how the intestinal commensal microbiome is able to communicate with the CNS through the immune pathway [113]. CD39 (NTPDase 1) is an immune regulatory enzyme with the ability to catalyze pro-inflammatory ATP into less-inflammatory ADP [114]. Whereas PSA has immunomodulation and anti-inflammatory activities, the data do not show its effect on AD neuroinflammation.

### 4.3 Neural pathway in the AD microbiota-gut-brain axis

The ENS is a major part of the autonomic nervous system and contains more than 100 million neurons. It forms a huge and complex nervous system, capable of controlling gastrointestinal physiology independent of the brain or the spine [115].

Enteric neurons are distributed in myenteric and submucosal ganglia, and the enteric glial cells and neuronal fibers travel the whole intestinal mucosa. In addition, ENS is a key regulator of permeability and function of IEB. The gut has rich innervation and contains more neurons than the spinal cord [116]. The structure and neurochemistry of the ENS are similar to the CNS, and thus the intestinal inflammation or gut dysbiosis may affect the brain's physiologic activities [117]. It was found that the GF mice lead to a reduction in enteric neurons and deficit in gut motility compared to the normal cells [118]. Oral administration of probiotic *Lactobacillus reuteri* showed increased excitability and decreased potassium channel opening rate in the enteric neurons [119]. Likewise, compared to the normal mice, the excitability of sensory neurons was decreased and the excitability of intestinal bacteria was restored in GF mice [120]. Gut microbiota also plays a vital role in modulating the glial cells in the intestinal mucosa, which are continuously renewed from the gut wall plexi [121].

To explore the potential role of ENS in AD, several studies demonstrated that progressive accumulation of Aβ in the brain was correlated with disease progression in enteric neurons in APP/PS1 transgenic mice, which was manifested as a decrease in the number of enteric neurons and the gut became more susceptible to inflammation [122,123]. In addition, Han et al. revealed that the accumulation of Aβ might activate the enteric resident macrophages, resulting in myenteric nitrergic and cholinergic neurons impairment in the ENS [124].

The vagus nerve (cranial nerve X) is another important neural pathway between the gut and brain in the microbiota-gut-brain axis. It plays both efferent and afferent roles in the communication between the gut and the brain. The vagus nerve comprises a major part of the sensory nerve, which conveys information from distant organs to the CNS. The CNS receives information tonically from the heart, lungs, stomach, and intestines via sensory fibers. Many studies have demonstrated that the vagus nerve plays an important role in the microbiota-gut-brain axis [58,62]. Many gut microbiota or probiotics have cognition modulatory effects dependent on the vagus nerve activity. For example, Bravo et al. revealed that, via the vagus nerve, *Lactobacillus rhamnosus* (*L. rhamnosus*) could modify the stress response, behaviors related to anxiety, depression, or cognition, as well as alter central levels of GABA receptors. On the other hand, there were no neurochemical and behavioral benefit effects associated with the *L. rhamnosus* in vasectomized mice [58].

The vagus nerve in the microbiota-gut-brain axis communicates directly through the vagal nerve pathway or indirectly through the vagal-nerve-mediated immunomodulation pathway [62,124]. For instance, in the direct route, Bercik showed that *B. longum* NCC3001 could normalize the anxiety-like behavior in gut chronic inflammation mice by innervating vagal afferent terminals, resulting in the upregulation of BDNF in the neural cells of the brain [62]. On the other hand, indirectly, vagal afferent signals from the intestines may instigate an anti-inflammatory reflex and activated efferent response, further resulting in the attenuation of inflammation in the brain [124]. Microbial amyloid not only exhibits pro-inflammatory effects but also influences the brain via the neural communication pathway.

In summary, both the ENS and the vagus nerve may interact with the brain directly, or indirectly, via the nerve-immunity crosstalk. Unfortunately, most of the ENS and vagus nerve studies in the microbiota-gut-brain axis have mainly focused on PD, depression, or anxiety. The data on how gut microbiota affects cognition and memory in AD mediated by ENS and vagus nerve remains scarce.

### 4.4 The microbiota-gut-brain axis targeted therapy in AD

Owing to the overwhelming evidence of the interaction between the gut microbiota and the development of AD, the microbiota-gut-brain axis has been considered as a potential target for drug development. We summarize currently the microbiota-gut-brain axis targeted therapy in AD, including carbohydrates or natural products, probiotics, and dietary ways, which can modulate the microbiota-gut-brain axis mainly through microbiota rebalance, and immune, metabolism pathway modulation in the AD microbiota-gut-brain axis.

Carbohydrates (monosaccharides, oligosaccharides, polysaccharides, and glycosides) are the primary source of nutrient for the microbiota and has a diverse modulatory effect on the microbiota [125]. Human milk oligosaccharides also showed a vital role in the guide and supporting the assembly of a healthy gut microbiome in infants [126]. GV-971 is an acidic linear marine-derived mixture of oligosaccharides, with a degree of polymerization from 2 to 10. It has completed a phase 3 clinical trial for AD treatment in China [30]. A Multicenter, randomized, double-blind phase 3 clinical trial also demonstrated significant efficacy of GV-971 in improving cognition across all observation periods of a 36-week trial [127]. In AD progression, immune cell infiltration and microglial activation occurred in the brain, resulting in neuroinflammation. It has been shown that GV-971 could ameliorate neuroinflammation by rebalancing the abnormal elevation of amino acids, phenylalanine, and isoleucine metabolism through the gut microbiota. Meanwhile, GV-971 also showed modulation in the immune pathway in the AD microbiota-gut-brain axis, which was characterized by increased peripheral infiltration of immune system cells (peripheral type 1 T-helper, Th1) to allow it local crosstalk with the M1 microglia and triggers the microglia differentiation toward an M1 pro-inflammatory state, thus highlighting the role of oligosaccharides in anti-neuroinflammation through modulating the microbiota metabolism and immune in the microbiota-gut-brain axis [30].

Besides, other studies have revealed that fructo-oligosaccharides and oligosaccharides from *Morinda officinalis* can ameliorate the learning and cognitive impairment in the AD model mice by reconditioning the diversity and stability of the microbial community [128,129]. For instance, Liuwei Dihuang decoction, a famous prescription in traditional Chinese medicine (TCM), contains polysaccharides, glycosides, and oligosaccharides. The oligosaccharide fraction derived from the *Liuwei Dihuang* decoction ameliorated the AD-like cognitive impairments through remodeling the gut microbiota in a neuroendocrine-immunomodulation pathway manner, in senescence-accelerated mouse prone 8 (SAMP8) [61]. In summary, there has been evidence that carbohydrates remodel the gut microbial composition and regulate host physiology and metabolism, presenting a huge opportunity for developing microbiota-gut-brain axis targeted medicine through the oligosaccharide-based microbiota composition rebalance and metabolism modulation approach (Table 3) [61,130]. Meanwhile, the oligosaccharides from TCM *Morinda officinalis* possess huge potential in gut microbiota modulation, suggesting its potential value in future drug development.

**Table 3:** Summary of the microbiota-gut-brain axis targeted therapy in AD

Drug categories	Drug	Effect	Experimental subject	References
Carbohydrates or natural products	Prebiotic fructooligosaccharides	Regulated the gut microbiota-GLP-1/GLP-1R pathway	APP/PS1 transgenic AD model mice	[138]
	Fructooligosaccharides from *Morinda officinalis*	Prebiotic effect; regulating the composition and metabolism of the gut microbiota	D-galactose- and Aβ1-42-induced deficient rats	[129]
	Oligosaccharides from *Morinda officinalis*	Regulated cholesterol, L-valine, and L-acetylcarnitine in serum	APP/PS1 transgenic AD model mice	[128]
	GV-971 (mixture of acidic linear oligosaccharides)	Regulating amino acid metabolism; alleviates neuroinflammation by shaping the gut microbiota	APP/PS1 transgenic AD model mice	[30]
	An oligosaccharide fraction derived from *Liuwei Dihuang* decoction	Alterations in the gut microbiota-neuroendocrine immunomodulation network; influenced the relative abundance of these intestinal microbiomes	SAMP8	[61]
	Camellia oil	Modulated the expression of immune-related cytokines by inhibiting RAGE/NF-κB signaling; enhanced autophagy; regulated microglial activation	SAMP8	[128]
	Sesamol	Improved the generation of microbial metabolites SCFAs; prevented gut barrier damages and systemic inflammation; improved synapse ultrastructure and inhibited Aβ accumulation	ApoE transgenic AD model mice	[139]
Probiotic	*Lactobacillus plantarum* DR7	Reduction in *Wolbachia*; an increase of *Stenotrophomonas* and *Acetobacter*	*Drosophila melanogaster* AD model	[140]
	*Bifidobacterium breve* strain A1	Metabolite acetate partially ameliorated the cognitive decline in AD mice; suppressed the hippocampal expressions of inflammation and immune-reactive genes	Intracerebroventricular injection of Aβ25–35 or Aβ1–42 induced AD model mice	[141]
	Probiotic supplementation (*Lactobacillus casei* W56, *Lactococcus lactis* W19, *Lactobacillus acidophilus* W22, *Bifidobacterium lactis* W52, *Lactobacillus paracasei* W20, *Lactobacillus plantarum* W62, *Bifidobacterium lactis* W51, *Bifidobacterium bifidum* W23, and *Lactobacillus salivarius* W24)	Increased in *Faecalibacterium prausnitzii*; activation of macrophages or dendritic cells	AD patients	[142]
	Probiotic-4 (*Bifidobacterium lactis*, *Lactobacillus casei*, *Bifidobacterium bifidum*, and *Lactobacillus acidophilus*)	Protective effect on IEB and BBB; inhibition of TLR4 and RIG-I-mediated NF-κB signaling pathway and inflammatory responses; seduced LPS in plasma and cerebral	SAMP8	[98]
	*Bifidobacterium longum*	Inhibited gut microbiota endotoxin production; reduced blood LPS levels; suppressed NF-*κ*B activation and TNF-α expression	5 × transgenic AD model mice	[99]
	Probiotic formulation (*Lactobacillus helveticus* R0052 and *Bifidobacterium longum* R0175)	Attenuated the decremental effect of LPS on memory through BDNF expression	Lipopolysaccharide induced neuroinflammation-associated disorders in the AD rat model	[23]
	*Streptococcus thermophilus*, *bifidobacteria* (*Bifidobacterium longum*, *B. breve*, *B. infantis*), *lactobacilli* (*Lactobacillus acidophilus*, *L. plantarum*, *L. paracasei*, *L. delbrueckii subsp. bulgaricus*, *L. brevis*)	Activated SIRT1 pathway; promoted antioxidant and neuroprotective effects	3 × transgenic AD model mice	[143]
	*Clostridium butyricum*	Protection against microglial-mediated neuroinflammation mediated by the metabolite butyrate; suppressed NF-*κ*B activation; inhibit the production of TNF and IL-1β in the brain	APP/PS1 Transgenic AD model Mice	[132]
	*Lactobacillus plantarum* MTCC1325	Ameliorated cognition deficits; restored Ach, and the histopathological features	D-Galactose-induced AD model mice	[144]
	Probiotic strains (*Lactobacillus plantarum NCIMB* 8826, *Lactobacillus fermentum* NCIMB 5221 and *Bifidobacteria longum* spp. *infantis* NCIMB 702255)	Metabolic stability, immune signaling, oxidative and mitochondrial stress through the gut-brain-axis	*Drosophila melanogaster* AD model	[145]
	*Bifidobacterium bifidum* BGN4 and *Bifidobacterium longum*	Improvement in mental flexibility test and stress score; relative abundance of gut bacteria reduced; increased serum BDNF level;	AD patients	[146]
Dietary ways	KD	Enhanced brain vascular and BBB function; increased beneficial gut microbiota; improved metabolic profile; reduced mTOR and increased eNOS protein expressions; increased *Akkermansia muciniphila* and *Lactobacillus* abundance; reduced *Desulfovibrio* and *Turicibacter* abundance	Mice	[147]
	Modified Mediterranean-ketogenic diet	Reduced fecal lactate and acetate while increasing propionate and butyrate; modulated the gut microbiome and metabolites in association with improved AD biomarkers in the cerebrospinal fluid	AD patients	[148]
	Dietary fiber	Interfering with the assembly of Aβ1-40 and Aβ1-42 peptides into soluble neurotoxic Aβ aggregates	*In vitro*	[149]
	Dietary inulin	Increased SCFAs, tryptophan-derived metabolites, bile acids, glycolytic metabolites, and scyllo-inositol in the gut; rebalanced the beneficial microbiota composition and host metabolism; reduced inflammatory gene expression in the hippocampus	APOE4 transgenic (E4FAD) mice	[70]
	Bioactive food	Abated neuroinflammation and oxidative stress; decreased astrocyte and microglial activation; restored the microbiota composition, LPS, and propionate levels in the gut	3 × Transgenic AD model mice	[150]
	MIND	Associated with a slower rate of cognitive decline	Study of volunteers living in retirement communities and senior public housing units	[135]

mTOR, mechanistic target of rapamycin; eNOS, endothelial nitric oxide synthase; BDNF, brain-derived neurotrophic factor; TLR4, toll-like receptor 4; RIG-I, retinoic-acid-inducible gene-I; LPS, lipopolysaccharides; IEB, intestinal epithelial barrier; BBB, blood–brain barrier; SCFAs, short-chain fatty acids; TNF, tumor necrosis factor; IL-1β, interleukin-1β.

Many recent studies have illustrated the diverse benefits of probiotic therapy potential in AD. Generally, probiotic exhibits anti-inflammatory and antioxidative effects in the AD microbiota-gut-brain axis against the detrimental effect of microbial secretory products [131]. *Bifidobacterium* and *Lactobacillus* are two major probiotic *genera* with beneficial effects on neurodegeneration diseases (Table 3). Specifically, *Bifidobacterium longum* can reduce LPS-stimulated NF-κB activation and inflammatory in the hippocampus [99]. Yang et al. demonstrated that probiotic-4 (*Bifidobacterium lactis*, *Lactobacillus acidophilus*, *Bifidobacterium bifidum*, and *Lactobacillus casei*) attenuated aging-related disruption of IEB and BBB, reduced LPS in plasma and cerebral, characterized by inhibition of the NF-κB inflammatory signaling pathway [98]. The studies elucidated that the two *genera* contain anti-inflammation effect on the NF-κB-induced immune response pathway. In addition, Sun et al. demonstrated that *Clostridium butyricum* attenuated microglia-mediated neuroinflammation, indexed by the TNF-α and IL1β expression in the brain, were reduced by butyrate [132]. Taken together, in the AD pathological state, probiotics can modulate the metabolism of gut SCFAs, alter the composition of the microbiota, and reduce the inflammatory responses mediated by the microbiota-gut-brain axis (Table 3). However, still, the limitations exist. Numerous reports have proposed the probiotic use to deleterious effects such as sepsis, immunoreactivity, and gene transfer resulting in pathogenic antibiotic resistance [133].

The ketogenic diet (KD), which is high fat and low carbohydrate diet, has been widely used as therapy for neurological disorders. The diet acts by increasing the beneficial gut microbiota as well as improving the metabolic profile (Table 3). On the other hand, another study showed that inulin, a dietary nondigestible carbohydrate fiber, can rebalance the beneficial microbiota composition and host metabolism in the cecum, periphery, and brain. Inulin increases the levels of SCFAs, scyllo-inositol, bile acids, glycolytic, and tryptophan metabolites [70]. Three closely related diets including Dietary Approaches to Stop Hypertension (DASH), Mediterranean diet, and Mediterranean-DASH diets, also called Mediterranean-DASH Intervention for Neurodegenerative Delay (MIND), were demonstrated to be linked to neuroprotection and dementia prevention and showed a slower rate of cognitive decline [134]. The effect of MIND was better than the Mediterranean diet and DASH [134,135]. Therefore, collectively, diet intervention or nutrition therapy that targets the microbiota-gut-brain axis plays a vital role in the microbiota-gut-brain axis through modulating gut microbiota metabolites (Table 3).

## 5 Conclusions and perspectives

**Figure 2:**
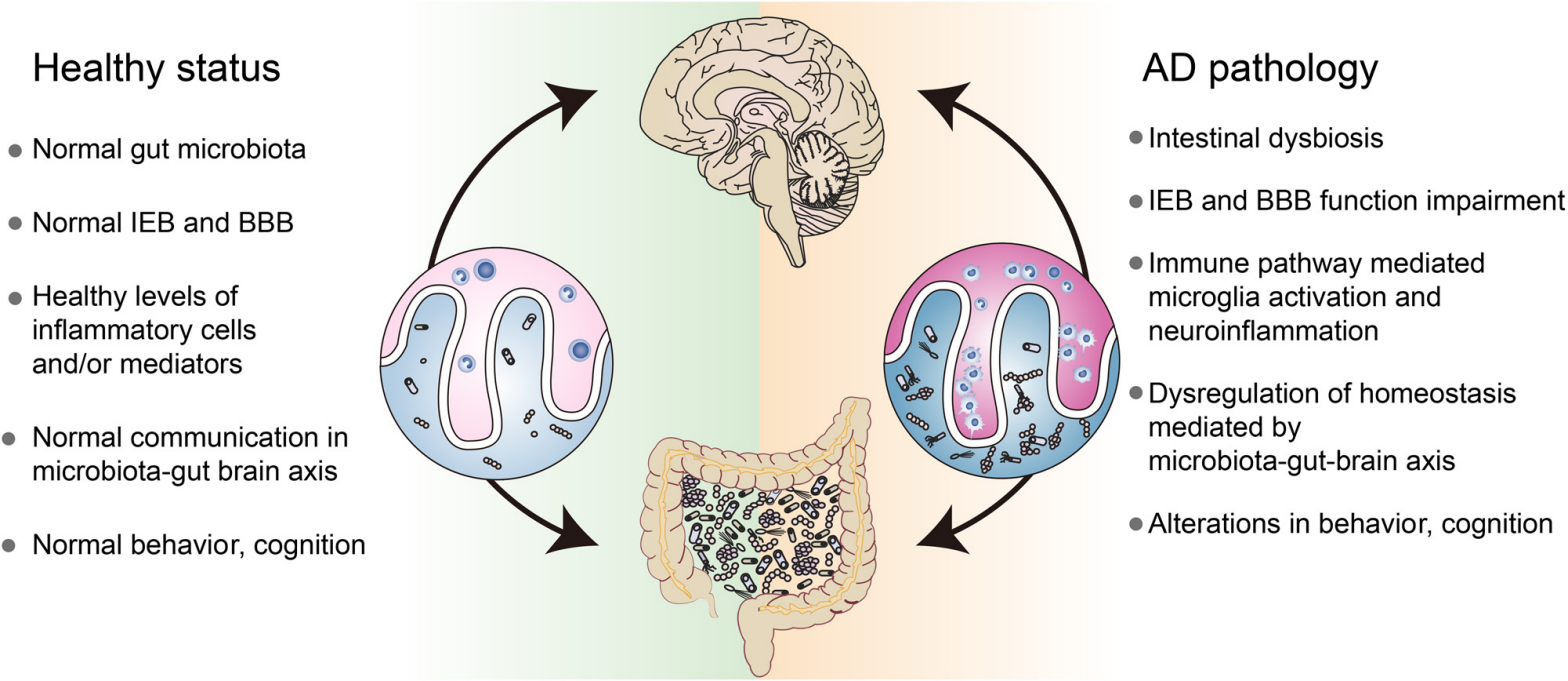
Impact of the gut microbiota on the microbiota-gut-brain axis in health and AD. It is well established that stable gut microbiota is the foundation for normal gut physiology and participate in heath signaling along the microbiota-gut-brain axis (right-hand side of this figure). The intestinal dysbiosis cascade and inappropriate microbiota-gut-brain axis signaling (right-hand side of this figure), just like the amyloid cascade hypothesis, oxidative stress, and genetic factors, have increasingly become a major research interest in AD pathogenesis. Meanwhile, the intestinal dysbiosis cascade may be a tractable target for AD drug development.

Microbes and other organisms co-evolve in a process referred to as symbiosis (such as legume-rhizobia symbiosis, sometimes even displayed as a genetic material exchange) or mutual exclusion (such as penicillin inhibit competitors, exhibit as natural selection and survival of the fittest). This biological relationship highlights the beneficial or harmful effects of gut microbiota in human physiological and pathological events. The data on the interplay between the intestines and CNS in AD pathogenesis is still limited. Here, we illustrated the communication between gut microbiota and the brain (the microbiota-gut-brain axis), and its potential roles in AD pathology (Figure 2).

Gut microbiota can interact with CNS through the microbiota-gut-brain axis via microbial secretions, metabolic intervention, and neural stimulation. In addition, we summarize the regulatory pathways of the microbiota-gut-brain axis such as the immune, neural, neuroendocrine, or metabolic pathways. We also highlight the significant roles of IEB and BBB in the microbiota-gut-brain axis. In AD pathogenesis, there is progressive perturbation between the gut microbiota and host co-metabolic relationship. This shift leads to gut dysbiosis and secretion of microbial products and metabolites, which may affect the CNS metabolic state and immune balance through the microbiota-gut-brain axis. It is well established that LPS stimulates the CNS innate immune response and microglial activation through the immune pathways. In fact, PSA has been associated with the migration of T regulatory cells to the CNS. However, its potential roles in AD remain elusive.

Jiang et al. have discussed the gut microbiota and AD [136]. They have provided a comprehensive overview of the connection between AD and gut microbiota including experimental evidence, microbiota-gut-brain axis, and highlighted the potential role of bacterial secretion in AD.

However, they mainly focused on the evidence that gut microbiota impacts AD, but without systematic elaboration on how the microbiota affects AD pathogenesis, including classification on the regulatory role of the immune and endocrine pathway. Moreover, the relationship between pathogenesis and microbiota is still uncertain: Is the microbiota change the cause or, instead, is it just a consequence of lifestyle, or other uncertain factor changes associated with the disease? Can it become a drug target to treat AD or other neurodegenerative diseases that need to be further studied?

Regardless, so far, the microbiota-gut-brain axis targeted treatment opens a new avenue for neurodegenerative disease therapy. Moreover, GV-971 has been shown to work by regulating gut microbiota dysbiosis, alleviating neuroinflammation, and ameliorating intestinal metabolism. It has not only unveiled the efficacy of the microbiota-gut-brain axis targeted AD therapeutic strategy but also provide a huge opportunity on novel potential drug candidates, the carbohydrates [30]. Carbohydrates form the primary nutrient source for microbiota and have a wide spectrum of modulatory effects on the microbiota [125]. There is, therefore, a need to interrogate the huge chemical space in carbohydrates. Accumulating evidence has robustly demonstrated that carbohydrates from diverse sources such as natural products, TCM, and marine-derived products, have a significant effect on remodeling gut metabolism and rebalancing the microbiota (Table 3). In addition, oligosaccharides derived from TCM have antineoplastic, neuroprotective, antidiabetic effects [137]. TCM might provide valuable resources for future development of the microbiota-gut-brain axis targeted treatment, as TCM theory always suggest that treat disease holistically. The concept of the microbiota-gut-brain axis suggests that remodeling the gut microbiota may be a tractable and novel therapeutic strategy for AD and other neurodegenerative diseases. Diet therapy also proposes a whole new way of preventing chronic or other neurodegenerative diseases through remodeling the gut progressively.
